# Ethylene Signal Is Involved in the Regulation of Anthocyanin Accumulation in Flesh of Postharvest Plums (*Prunus salicina* Lindl.)

**DOI:** 10.3390/plants12040893

**Published:** 2023-02-16

**Authors:** Xueling Li, Yudou Cheng, Ying Wang, Xiaohe Yang, Chuangqi Wei, Junfeng Guan

**Affiliations:** 1 Institute of Biotechnology and Food Sciences, Hebei Academy of Agriculture and Forestry Sciences, Shijiazhuang 050051, China; 2 Plant Genetic Engineering Center of Hebei Province, Shijiazhuang 050051, China

**Keywords:** plum, ethylene, anthocyanin accumulation, gene expression

## Abstract

Ethylene is positively correlated with the anthocyanin accumulation in postharvest plum fruit, but the regulation mechanism has not been fully clarified. In this work, the ‘Friar’ plum fruit under different storage temperatures (0, 10 and 25 °C) and treatments (100.0 μL L^−1^ ethylene and 1.0 μL L^−1^ 1-MCP) were applied to study the relationship between anthocyanin accumulation and ethylene signal pathway. The fruits stored at 10 °C had higher ethylene production rate and more anthocyanin in flesh than those stored at 0 °C and 25 °C. Ten ethylene biosynthesis associated genes and forty-one ethylene signal transduction related genes were obtained from the previous transcriptome data. Among them, the expression levels of ethylene biosynthesis associated genes (*PsACS1*, *PsACS4* and *PsACO1*), and ethylene signal transduction related genes (*PsERS1s*, *PsETR2*, *PsERF1a*, and *PsERF12*) were markedly higher in the fruits stored at 10 °C than those at 0 °C and 25 °C. Exogenous ethylene treatment enhanced while 1-MCP treatment inhibited the anthocyanin accumulation in the flesh under storage at 10 °C. In addition, exogenous ethylene treatment markedly increased the expression levels of *PsACS1*, *PsACS4*, *PsACO1*, *PsETR2*, *PsERF1a*, and *PsERF12* in the flesh once it turning red, as well as the anthocyanin biosynthesis related genes (*PsPAL*, *PsCHS*, *PsF3H*, *PsDRF*, *PsANS*, *PsUFGT* and *PsMYB10*), whereas 1-MCP treatment manifested the contrary effects. Correlation analysis indicated that there was a significant positive correlation between genes expression related to ethylene signal pathway and anthocyanin biosynthesis, except for *PsERF11*. In conclusion, ethylene signal pathway is involved in the flesh reddening by up-regulating the anthocyanin biosynthesis related genes.

## 1. Introduction

Ethylene plays an important role in fruit ripening. The initial step of ethylene biosynthesis in higher plants is the conversion of methionine into S-adenosyl methionine (SAM) catalyzed by adenosylmethionine synthase. SAM is carboxylated to 1-aminocyclopropane-1-carboxylic acid (ACC) by 1-aminocyclopropane-1-carboxylic acid synthase (ACS), and then the ACC is oxidized by 1-aminocyclopropane-1-carboxylic acid oxidase (ACO), subsequently producing ethylene [1]. Ethylene is perceived by five membrane-bound receptors, such as ethylene receptor 1 (ETR1), ethylene receptor 2 (ETR2), ethylene-responsive sensor 1 (ERS1), ethylene-responsive sensor 2 (ERS2) and ethylene insensitive 4 (EIN4). Raf-like kinase constitutive triple response 1 (CTR1) is the next downstream component in this pathway, and it is considered as a negative regulator to suppress the activation of downstream factor ethylene insensitive 2 (EIN2) by phosphorylation [2,3]. Once EIN2 is dephosphorylated, its C-terminal will be cleaved and then get into the cell nucleus to activate transcription factors ethylene insensitive 3 (EIN3) and ethylene insensitive 3-like (EIL) [4,5,6,7]. EIN3/EILs are the positive regulators and they can directly activate the expression of ethylene response factors (ERFs). The ERFs are considered as the last regulatory factors of ethylene signal transduction, and it can bind and regulate the genes in which promoter region contain the GCC-box *cis*-acting element, thus inducing ethylene response [8,9,10].

It has been known that ethylene is associated with anthocyanin biosynthesis. In *Arabidopsis*, ethylene treatment inhibited the anthocyanin accumulation by down-regulating the transcription factors TRANSPARENT TESTA 8 (AtTT8) and PRODUCTION OF ANTHOCYANIN PIGMENT1 (AtPAP1) that related to anthocyanin biosynthesis [11]. It is found that exogenous ethylene treatment delays the anthocyanin accumulation and subsequently color development, but 1-methylcyclopropene (1-MCP, an ethylene action antagonist) treatment promotes the fruit reddening and enhances the anthocyanin content in peach [12]. In ‘Hongzaosu’ pear, the ethylene-activated transcription factor PpERF105 inhibited the anthocyanin accumulation by inducing the expression of the R2R3-MYB type transcription factor PpMYB140, while the latter was known as a suppressor of anthocyanin biosynthesis [13]. However, more studies focus on the positive regulation of ethylene on anthocyanin biosynthesis. It has been confirmed that ethylene improved red color by up-regulating the expression levels of anthocyanin biosynthesis related genes in the grape and plum fruits [14,15]. In apples, the transcription factor MdEIL1 promoted anthocyanin accumulation by inducing the expression of *MdMYB1* (a key positive regulator for anthocyanin biosynthesis), and MdERF1B and MdERF38 interacted with different R2R3-MYB transcription factors to positively regulate anthocyanin biosynthesis [16,17,18]. In addition, several ERF/AP2 transcription factors, such as PyERF3 [19], PbERF22 [20], Pp4ERF24 and Pp12ERF96 [21], also have been proved to positively regulate anthocyanin biosynthesis in pears. In summary, it is indicated that the ethylene signal pathway plays an important role in regulating anthocyanin biosynthesis [11], but the mechanism may be distinct in different species.

Red appearance is an important quality indicator that affects the commodity value of fruits. Therefore, the anthocyanin biosynthesis pathway and its regulating mechanism are widely studied. However, most of the reports focus on the color development of peel under light condition [22,23,24], while it is still unclear about the mechanism of anthocyanin accumulation in flesh in which the red color development did not require light induction. Previous studies have shown that the moderate temperature storage can significantly promote the anthocyanin accumulation in the flesh of ‘Friar’ plum fruit [25,26,27], but the mechanism about the involvement of ethylene signal is little known. So, in this work, the candidate genes related to ethylene signal pathway were firstly screened based on the previous transcriptome analysis [27], and then their expression patterns in the flesh of ‘Friar’ plum fruit under different temperature conditions and ethylene/1-MCP treatments were observed. Further, the relationship between ethylene signal pathway and anthocyanin biosynthesis was analyzed to reveal the mechanism that the moderate temperature storage promoted flesh reddening in postharvest ‘Friar’ plum fruit.

## 2. Results

### 2.1. Changes of the Anthocyanin Content and Ethylene Production Rate under Different Storage Temperatures

**Figure 1:**
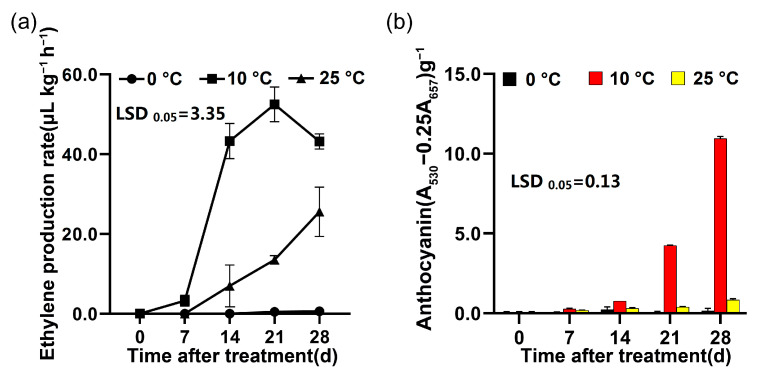
Changes of ethylene production rate (**a**) and anthocyanin content in flesh (**b**) of ‘Friar’ plum fruit under different storage temperatures. The vertical bar represents the standard error while the LSD values show significant differences at the 0.05 level.

Under different storage temperatures (0, 10 and 25 °C), there were significant differences in ethylene production rate and anthocyanin content in the flesh of ‘Friar’ plum fruit. When the fruit was stored at 25 °C, the ethylene production rate increased gradually, while it remained at a low level in the fruit under storage at 0 °C. Differently, the ethylene production rate increased significantly and reached its peak on the day 21 of storage, and presented the highest level during storage at 10 °C (Figure 1a). Meanwhile, the anthocyanin content in the flesh stored at 10 °C obviously increased on day 14, and was significantly higher than those at 25 °C and 0 °C (Figure 1b).

### 2.2. Expression of Candidate Genes Associated with Ethylene Biosynthesis in Flesh of ‘Friar’ Plum Fruit under Different Storage Temperatures

**Figure 2:**
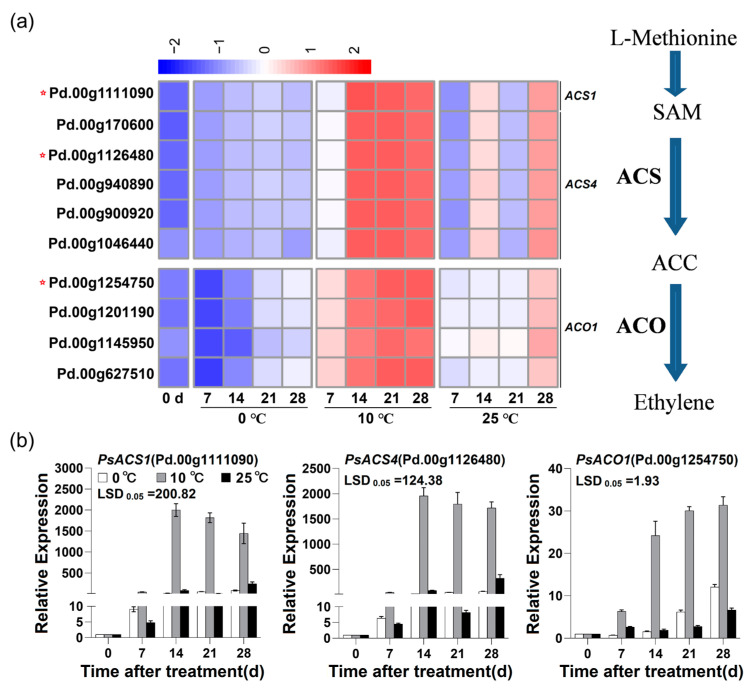
Expression profiles of *PsACSs* and *PsACO1s* in the flesh of Friar plum fruit under different storage temperatures. (**a**) Heatmap of the expression levels of candidate genes associated with ethylene biosynthesis via transcriptome analysis; red stars labeled gene IDs represent the genes detected by qRT-PCR in (**b**). (**b**) qRT-PCR detection of *PsACS1*, *PsACS4* and *PsACO1*. The vertical bar represents the standard error while the LSD values show significant differences at the 0.05 level.

Based on our previous transcriptome analysis, six differentially expressed *ACS* genes in the flesh of ‘Friar’ plum fruit under different storage temperatures were identified and named as *PsACS1* and *PsACS4s*, respectively. Interestingly, the expression patterns of these six *ACSs* in the flesh stored at 10 °C were markedly higher than those at 0 °C and 25 °C. In addition, four differentially expressed *ACO1* genes were detected. Similar to *ACSs*, the four *ACO1* genes showed the highest expression level in the flesh stored at 10 °C (Figure 2a). Considering the similar expression patterns of the homologous genes and their high identity in nucleic acid sequences (Supplementary Table S1), the *PsACS1*, *PsACS4* and *PsACO1* which labeled with red stars in Figure 2a were selected for qRT-PCR verification. The result of qRT-PCR assay indicated that the three selected genes showed obviously higher expression level in the flesh stored at 10 °C (Figure 2b), and this pattern was consistent with the analysis of differentially expressed genes (DEGs) based on transcriptome data.

### 2.3. Expression of Candidate Genes Associated with Ethylene Signal Transduction in Flesh of ‘Friar’ Plum Fruit under Different Storage Temperatures

**Figure 3:**
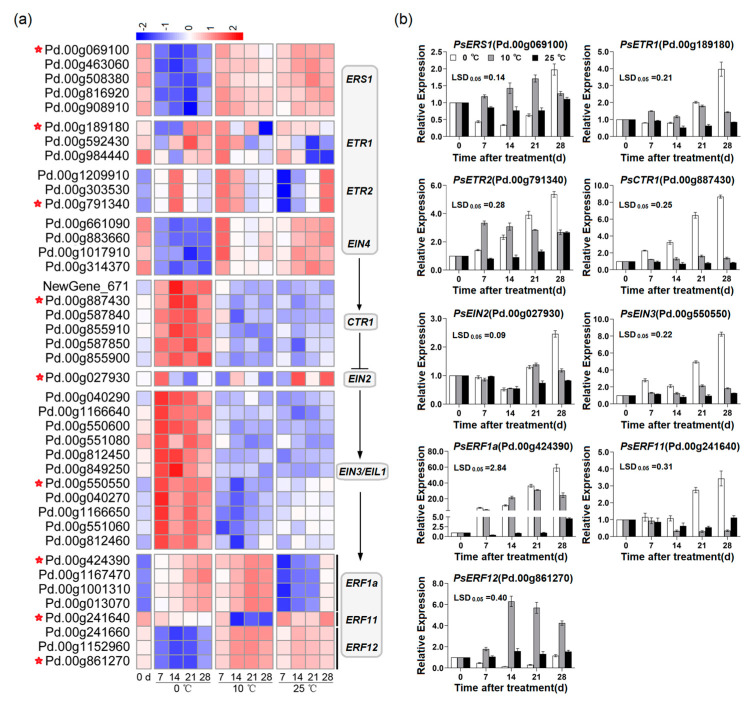
Expression profiles of ethylene signal transduction related genes in flesh of ‘Friar’ plum fruit under different storage temperatures. (**a**) Heatmap of the expression levels of candidate genes related to ethylene signal transduction; The red stars labeled gene IDs represent the genes detected by qRT-PCR in (**b**). (**b**) qRT-PCR detection of the selected genes. The vertical bar represents the standard error while the LSD values show significant differences at the 0.05 level.

Forty-one DEGs related to ethylene signal transduction were selected based on the transcriptome analysis. Fifteen candidate ethylene receptor genes in the flesh stored at 10 °C (*ERS1*, *ETR1*, *ETR2* and *EIN4*) showed significantly higher expression levels than those stored at 25 °C and 0 °C on day 7, while four *ERF1a*, and three *ERF12* in the flesh stored at 10 °C showed the higher expression level than those stored at 25 °C and 0 °C on days 14 and 21 (Figure 3a).

Similar to the ethylene biosynthesis related genes, there were multiple gene identifications corresponding to the same gene in the ethylene signal transduction, and these homologous genes showed similar expression patterns and high identity of nucleic acid sequences (Supplementary Table S1). So, the genes *PsERS1*, *PsETR1*, *PsETR2*, *PsCTR1*, *PsEIN2*, *PsEIN3*, *PsERF1a*, *PsERF11*, and *PsERF12* which labeled with red stars in Figure 3a were explored for qRT-PCR verification and subsequent analysis in this work. 

The qRT-PCR assay indicated that the expression level of *PsERS1* in the flesh stored at 10 °C was significantly higher than those stored under 25 °C and 0 °C on days 7, 14 and 21. Similarly, *PsETR1* and *PsETR2* in the flesh stored at 10 °C showed the higher expression level on days 7 and 14. Under storage at 10 °C, the expression levels of *PsCTR1* and *PsEIN3* in flesh were significantly higher than those at 25 °C, but lower than those under storage at 0 °C. The expression level of *PsEIN2* in the flesh stored at 10 °C was obviously higher than that stored at 25 °C on days 21 and 28, while lower than that stored at 0 °C on day 28. In the flesh stored at 10 °C, the expression level of *PsERF1a* increased sharply and was higher than that stored at 25 °C, while it was significantly lower than that stored at 0 °C on day 28. After 14 days of storage, the mRNA amount of *PsERF11* in the flesh stored at 10 °C was lower than those stored at 25 °C and 0 °C. The fruit stored at 10 °C showed increasing transcripts of *PsERF12* on days 14, 21 and 28, which were markedly higher than those at 25 °C and 0 °C (Figure 3b). The expression profiles of the above-mentioned genes observed by qRT-PCR analysis were similar to the result of DEGs.

### 2.4. Effects of 1-MCP and ETH on the Anthocyanin Accumulation in the Flesh of ‘Firar’ Plum under Storage at 10 °C

**Figure 4:**
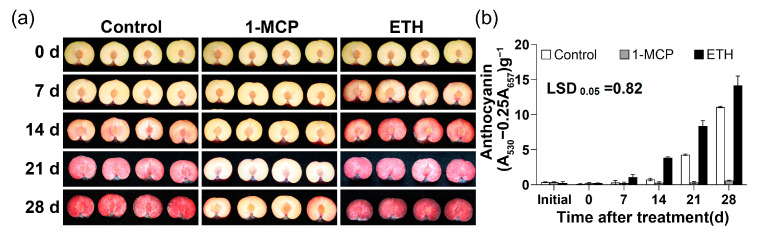
Effects of 1-MCP (1.0 μL L^−1^) and ETH (100.0 μL L^−1^) treatments on the changes of flesh color (**a**) and anthocyanin content (**b**) of ‘Friar’ plum fruit stored at 10 °C. “Initial” represents the time point before ethylene and 1-MCP treatments. The image of fruits on initial day is omitted in (**a**) and no obvious difference with that on day 0. The vertical bar represents the standard error while the LSD values show significant differences at the 0.05 level.

To further confirm the regulation of ethylene on anthocyanin biosynthesis, the effects of ETH and 1-MCP treatment on the flesh anthocyanin accumulation of ‘Friar’ plum fruit were studied. It showed that ETH treatment could promote the flesh reddening, while 1-MCP treatment had the opposite effect (Figure 4a). Moreover, the content of anthocyanin in the flesh of ‘Friar’ plum fruit treated with ETH increased after 7 days of treatment, and it was significantly higher than control. On the contrary, 1-MCP treatment markedly inhibited anthocyanin accumulation (Figure 4b).

### 2.5. Effects of 1-MCP and ETH on the Expression of Genes Associated with Ethylene Biosynthesis and Signal Pathway in the Flesh of ‘Friar’ Plum under Storage at 10 °C

**Figure 5:**
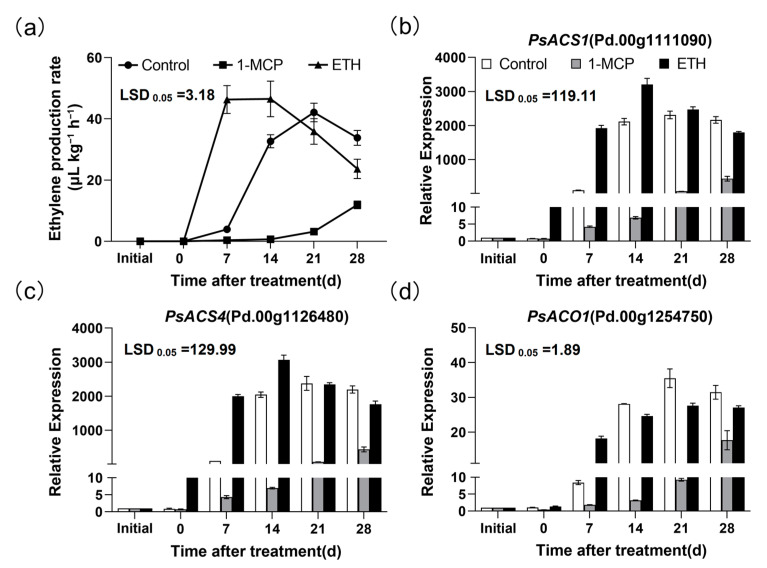
Effects of 1-MCP (1.0 μL L^−1^) and ETH (100.0 μL L^−1^) treatments on the ethylene production rate (**a**) and expression profiles of genes associated with ethylene biosynthesis (**b**–**d**) in ‘Friar’ plum fruit under 10 °C storage. “Initial” represents the time point before ethylene and 1-MCP treatments. The vertical bar represents the standard error while the LSD values show significant differences at the 0.05 level.

The ethylene production rate reached the peak earlier in the ETH-treated ‘Friar’ plum fruit, and was significantly higher than control group after 7 and 14 days of treatment, while it was much lower in 1-MCP-treated fruit (Figure 5a). Further study indicated that the expression levels of *PsACS1* and *PsACS4* in ETH-treated fruit were significantly higher than control group after 0, 7 and 14 days of treatment. In contrast, 1-MCP treatment lowered the expression levels of *PsACS1* and *PsACS4* (Figure 5b,c). Compared with control group, the transcript of *PsACO1* of ETH-treated fruit was higher after 7 days of treatment, but lower after 14, 21 and 28 days of treatment. However, the expression level of *PsACO1* in the 1-MCP treated fruit was lower than that of the control group after treatment (Figure 5d).

**Figure 6:**
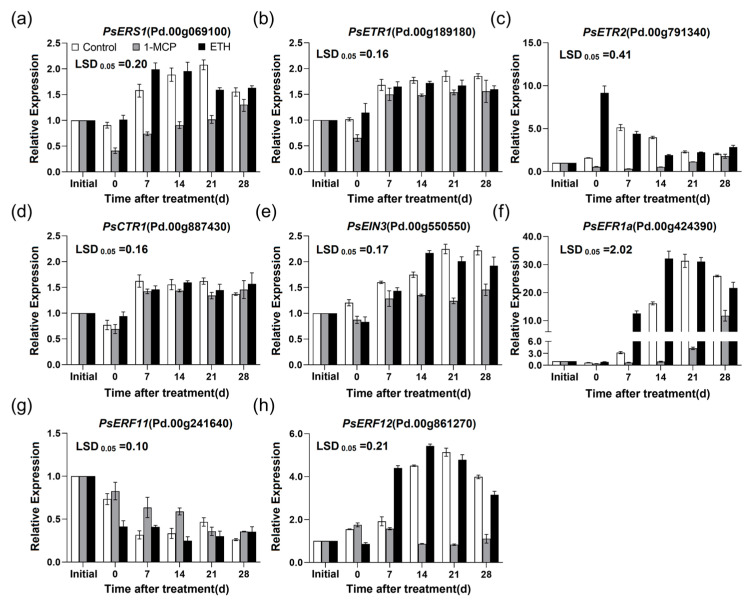
Effects of 1-MCP (1.0 μL L^−1^) and ETH (100.0 μL L^−1^) treatments on the expression profiles of genes associated with ethylene signal transduction in ‘Friar’ plum fruit under 10 °C storage. (**a**–**h**) Relative expression of *PsERS1*, *PsETR1*, *PsETR2*, *PsCTR1*, *PsEIN3*, *PsERF1a*, *PsERF11* and *PsERF12*. “Initial” represents the time point before ethylene and 1-MCP treatments. The vertical bar represents the standard error while the LSD values show significant differences at the 0.05 level.

Furthermore, the expression patterns of ethylene signal transduction related genes were also different in the ‘Friar’ plum fruit under 1-MCP and ETH treatments. In the ETH-treated ‘Friar’ plum fruit, the expression level of *PsERS1* increased significantly after 7 days of treatment and was higher than that in control, while they were decreased in the 1-MCP treated-fruit (Figure 6a). ETH treatment did not enhance the expression level of *PsETR1*, but 1-MCP treatment obviously decreased it (Figure 6b). Compared with control group, the transcript of *PsETR2* was markedly up-regulated after ETH treatment, while down-regulated by 1-MCP treatment until 21 days after treatment (Figure 6c). There was nearly no significant difference in the expression pattern of *PsCTR1* among the treatments (Figure 6d). For *PsEIN3*, ETH treatment increased its mRNA amount slightly on the 14th day after treatment, while 1-MCP treatment inhibited it from 14 to 28 days after treatment (Figure 6e). The expression levels of *PsERF1a* and *PsERF12* in ETH-treated fruit were markedly higher than that in control after 7 and 14 days of treatment. On the contrary, they were lower in 1-MCP-treated fruit (Figure 6f,h). Differently, the expression level of *PsERF11* gradually decreased in all treatments, and ETH treatment accelerated this process, while 1-MCP was opposite (Figure 6g).

### 2.6. Effects of 1-MCP and ETH on the Expression of Genes Associated with Anthocyanin Biosynthesis in the Flesh of ‘Friar’ Plum under Storage at 10 °C

**Figure 7:**
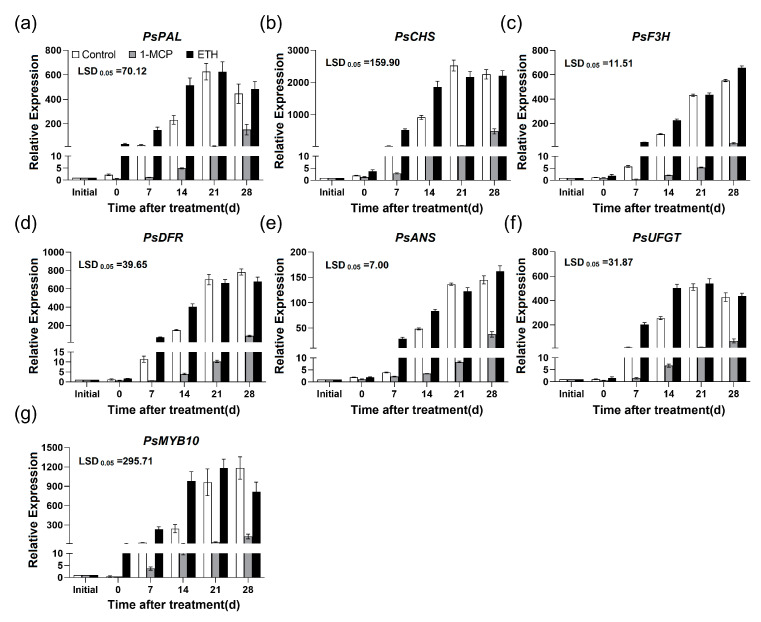
Effects of 1-MCP (1.0 μL L^−1^) and ETH (100.0 μL L^−1^) treatments on the expression profiles of genes associated with anthocyanin biosynthesis in ‘Friar’ plum fruit under 10 °C storage. (**a**–**g**) Relative expression of *PsPAL*, *PsCHS*, *PsF3H*, *PsDFR*, *PsANS*, *PsUFGT* and *PsMYB10*. “Initial” represents the time point before ethylene and 1-MCP treatments. The vertical bar represents the standard error while the LSD values show significant differences at the 0.05 level.

Compared with control group, ETH treatment significantly enhanced the transcripts of the structural genes involved in anthocyanin biosynthesis (*PsPAL*, *PsCHS*, *PsF3H*, *PsDRF*, *PsANS* and *PsUFGT*) at the earlier stage of the flesh reddening, while 1-MCP treatment markedly inhibited them (Figure 7a–f). At the same time, the regulating gene *PsMYB10* was also induced by ETH treatment. On the contrary, it was depressed by 1-MCP treatment (Figure 7g).

### 2.7. Correlation Analysis of Detected Factors

**Figure 8:**
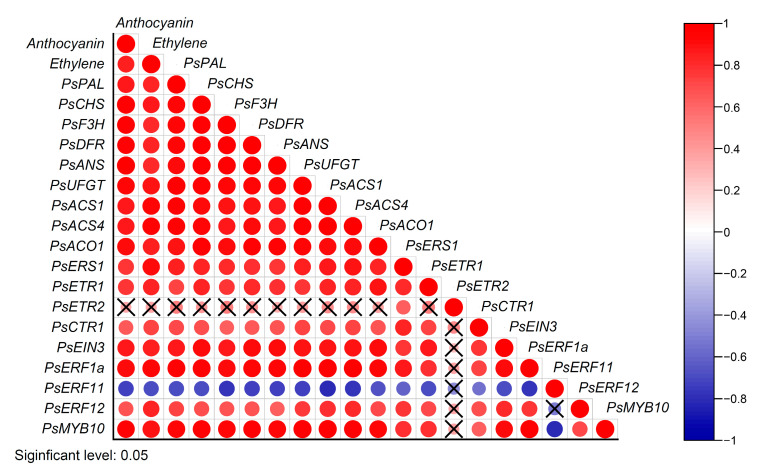
Correlation analysis among ethylene production rate, anthocyanin content, expression of genes associated with ethylene signal pathway and anthocyanin biosynthesis. n = 21, × represents no significant correlation between the two sets of data.

To get a better insight of the mechanism about ethylene-mediated flesh reddening, the correlation analysis of ethylene production rate, anthocyanin content, and expression patterns of genes related to ethylene signal pathway and anthocyanin biosynthesis was also studied in this work. There was a significant positive correlation between the change of anthocyanin content and ethylene production rate. Except *PsETR2*, the expression patterns of candidate ethylene signal pathway related genes such as *PsACS1*, *PsACS4*, *PsACO1*, *PsERS1*, *PsETR1*, *PsCTR1*, *PsEIN3*, *PsERF1a* and *PsERF12* were positively correlated to ethylene production rate, anthocyanin content and anthocyanin biosynthesis related genes, while *PsERF11* was negatively correlated (Figure 8). These results further indicated that the ethylene signal pathway was involved in regulating the anthocyanin accumulation in the flesh of postharvest ‘Friar’ plum.

## 3. Discussion

It has been found that the red development was accompanied by endogenous ethylene production in apple and plum fruits, and exogenous ethylene treatment could enhance anthocyanin accumulation of peel [15,16,28,29]. In this work, the ethylene production rate significantly increased in the ‘Friar’ plum fruit stored at 10 °C (Figure 1a). At the same time, the anthocyanin content of flesh sharply accumulated (Figure 1b), which was similar to previous study in ‘Aozhou 14’ plum fruit [30]. Further study indicated that it promoted the expression level of genes related to ethylene biosynthesis (*PsACS1*, *PsACS4* and *PsACO1*) under storage at 10 °C (Figure 2), suggesting that storage at 10 °C might induce flesh reddening of ‘Friar’ plum fruit by positively regulating ethylene production. Moreover, exogenous ethylene treatment increased the anthocyanin accumulation and the expression of genes related to anthocyanin biosynthesis and ethylene biosynthesis, while 1-MCP decreased (Figure 5 and Figure 7). Thus, it further indicates that the ethylene signal is involved in regulating the anthocyanin biosynthesis in flesh of postharvest ‘Friar’ plum fruit.

As a molecular switch of ethylene signal transduction, ethylene receptors are the key factors of ethylene-regulated biological process. In this study, the expression profiles of *PsETR1* and *PsERS1* were positively correlated with ethylene production and anthocyanin accumulation, as well as with the anthocyanin biosynthesis-related genes (Figure 8). These results were similar to the findings in ‘Red d’Anjou’pear and ‘Oishi-wase’ plum [15,31], thus it suggested that *PsETR1* and *PsERS1* were involved in ethylene-regulated anthocyanin accumulation in the flesh of postharvest ‘Friar’ plum fruit. The expression pattern of *PsETR2* was not correlated with ethylene production and anthocyanin accumulation (Figure 8), but similar to *PsETR1* and *PsERS1*, its expression level was up-regulated by moderate temperature storage (10 °C) and ETH treatment, and down-regulated by 1-MCP treatment. So, it was proposed that *PsETR2* also participated in ethylene-mediated flesh reddening of postharvest ‘Friar’ plum fruit. Although the expression pattern of *PsCTR1* was positively correlated with ethylene production, anthocyanin accumulation and the expression profiles of anthocyanin biosynthesis related genes (Figure 8), it did not show significant changes for the expression level once the flesh turning red (Figure 3), and was not affected by ethylene and 1-MCP treatment (Figure 6), suggesting that *PsCTR1* might not be the key gene to regulate anthocyanin accumulation in the flesh of postharvest ‘Friar’ plum fruit.

EIN3/EIL1 is an important transcription factor that initiates the downstream transcription induced by ethylene, and its function in anthocyanin biosynthesis is well known. In apple, MdEIL1 has been proposed to bind the promoter of *MdMYB1* and subsequently activate its expression, thus promoting anthocyanin accumulation under light condition. At the same time, the transcription factor MdMYB1 activates the expression of *MdERF3* that positively regulates ethylene biosynthesis, and then forms a feedback regulation cycle of ethylene-mediated anthocyanin biosynthesis [16]. In this study, the transcripts of screened *EIN3/EIL1s* did not significantly increase at the earlier stage of reddening in the flesh of ‘Friar’ plum fruit stored at 10 °C (Figure 3). Moreover, at the initial stage of flesh turning red in ETH-treated fruit, the expression level of *PsEIN3* was not significantly up-regulated. Thus, *PsEIN3* might not be involved in regulating anthocyanin accumulation in the flesh of ‘Friar’ plum fruit, and it is different from the red development of peel induced by light.

Recently, the function of ERF in regulating anthocyanin biosynthesis of fruit is also well studied. Over-expression the *JcERF035* gene from physic put in *Arabidopsis* can enhance the phosphate starvation-induced anthocyanin accumulation [32]. In apple, MdERF38 and MdERF1B can interact with different MdMYBs to regulate the light-induced anthocyanin biosynthesis [17,18]. Moreover, Pp4ERF24 and Pp12ERF96 have been known to regulate the blue light-induced anthocyanin accumulation by interacting with PpMYB114, and PyERF3 interacts with PyMYB114 and PybHLH3 to regulate anthocyanin biosynthesis in ‘Red Zaosu’ pear [19,21]. These findings imply that the ERFs may regulate anthocyanin biosynthesis through diverse pathways. In this work, the two selected ERF genes, *PsERF1a* and *PsERF12* were induced by exogenous ethylene and inhibited by 1-MCP, and their expression patterns were coordinated with those of genes related to anthocyanin biosynthesis (Figure 6 and Figure 7). Therefore, it is proposed that *PsERF1a* and *PsERF12* are involved in the moderate temperature-induced anthocyanin biosynthesis of ‘Friar’ plum fruit, as to their specific regulating function on anthocyanin biosynthesis related structural and regulatory genes (such as *PsMYB10*), it needs further study. Previous study indicates that MaERF11 inhibits the expression of *MaACO1* gene by recruiting histone deacetylase MaHDA1, thus delaying the ethylene-mediated fruit senescence in banana [10]. In this work, the *PsERF11* showed decreased expression level while the ethylene production rate and ethylene biosynthesis related genes increasing, indicating *PsERF11* might be a negative factor of ethylene biosynthesis regulation. Therefore, it is suggested that *PsERF11* might negatively regulate anthocyanin biosynthesis by inhibiting endogenous ethylene production.

## 4. Materials and Methods

### 4.1. Materials and Treatments

‘Friar’ (*Prunus salicina* Lindl.) plum fruit were harvested at commercial maturity (average single fruit weight: 117.00 ± 8.43 g; soluble solids content: 11.01 ± 0.13% and firmness: 70.36 ± 6.90 N) from an orchard located in Yixian county, Hebei Province, China (39°02′–39°35′ N, 114°51′–115°37′ E), and then immediately transported to the laboratory within 2 h on August 11, 2018. The fruit with uniform weight and shape, without visible defects were selected as materials, and the treatments were carried out as follows:

For different temperatures storage: The fruit were randomly divided into three groups (each about 350 fruit), and then were directly stored at 25 ± 1 °C (25 °C), 10 ± 0.5 °C (10 °C), and 0 ± 0.5 °C (0 °C), respectively. The ethylene production rate and anthocyanin content were observed at 0, 7, 14, 21, and 28 days of storage, and then the flesh samples were quickly frozen in liquid nitrogen and stored at −80 °C until used. Three replicates were performed for each treatment with 10 fruit per replicate.

For ethylene (ETH) and 1-methylcyclopropene (1-MCP) treatments: The plum fruit were randomly divided into three groups (each about 350 fruit). The fruit were carefully put into a sealed plastic box, and then were exposed to 100.0 μL L^−1^ ethylene and 1.0 μL L^−1^ 1-MCP at 25 ± 2 °C for 20 h, respectively. The group with air was set as control. After treatment, plum fruit were stored at 10 °C. The ethylene production rate and anthocyanin content were observed at initial (pre-treatment), 0, 7, 14, 21, and 28 days after treatment, and then the flesh samples were quickly frozen in liquid nitrogen and stored at −80 °C until used. Three replicates were performed for each treatment with 10 fruits per replicate.

### 4.2. Ethylene Production Rate Assay

Ten fruits of per replicate were sealed in the gas-tight containers (5.0 L) for 5 h. A 1.0 mL gas sample was selected and analyzed using a gas chromatograph (GC97902 II; Fuli Chromatograph Instruments Co., Zhejiang, China) equipped with a GDX-102 column and a flame ionization detector (FID). The temperatures of column, vaporization oven and FID were 78 °C, 120 °C and 200 °C, respectively. The carrier gas was N_2_ with a rate of 40 mL min^−1^. Ethylene production rate was expressed as μL kg^−1^ h^−1^. Three replicates were performed for each treatment.

### 4.3. Detection of Anthocyanin Content

About 0.3 g of frozen flesh tissue was homogenized by liquid nitrogen, and then was placed in 5 mL of 1% HCl in methanol (v/v) and subsequently incubated overnight in the dark at 4 °C. After centrifuging at 12,000× *g* for 5 min at 4 °C, the supernatant was selected and measured at 530 and 657 nm by a *UV-Vis* spectrophotometer (UV2600, SHIMADZU, Kyoto, Japan). The relative anthocyanin content was calculated using the equation A_530_ − 0.25 A_657_ and was represented as OD (A_530_ − 0.25 A_657_) g^−1^. Three replicates were performed for each treatment.

### 4.4. Analysis of Transcriptome 

Based on previous transcriptome data [27], the differentially expressed genes (DEGs) involved in ethylene biosynthesis and signal transduction (Fold Change ≥ 2 and FDR < 0.01) were selected according to the annotation information of KEGG pathway. The FPKM values of the selected DEGs were visualized as heat maps using the R language.

### 4.5. RNA Extraction and Quantitative Real-Time PCR (qRT-PCR) Analysis

Total RNA of flesh was extracted by the CTAB method [33]. After the electrophoretic analysis, 0.8 μg total RNA was applied for reverse transcription using the PrimeScript™ RT Reagent Kit with gDNA Eraser (Takara Bio Inc., Dalian, China). The qRT-PCR reaction was carried out in a final volume of 20.0 µL containing 10.0 µL SYBR Green PCR Premix Ex Taq™ (Takara Bio Inc., Dalian, China), 0.5 µL of ROX Reference DyeⅡ, 0.5 µL of forward and reverse primer (10 µmol L^−1^), 5 ng of cDNA and 6.0 µL of ultrapure water, and then was performed on an ABI 7500 instrument (Applied Biosystems, Foster City, CA, USA). The reaction procedure was as follows: 10 s at 95 °C, 40 cycles of 95 °C for 5 s, and 60 °C for 34 s. *PsACTIN7* was used to normalize the amount of gene-specific qRT-PCR products, and the relative expression levels of the detected genes were calculated according to the 2^−△△Ct^ method. The sequences of the qRT-PCR primers were shown in the Supplementary Table S2.

### 4.6. Statistical Analysis

Experimental results were analyzed using GraphPad Prism 8, IBM SPSS Statistics 23 and RStudio software. Error bars denote standard deviations. Least significant differences (LSD) at a significance level of 0.05 were generated by ANOVA using IBM SPSS Statistics 23. Correlation analysis and heat map figures were drawn by Origin 2021 software. Gene expression heat maps were drawn by Rstudio (pheatmap).

## 5. Conclusions

Ethylene signal positively regulates anthocyanin accumulation in the flesh of postharvest ‘Friar’ plum fruit. Moderate temperature storage promotes flesh turning red by up-regulating the expression of genes related to ethylene signal pathway and anthocyanin biosynthesis. *PsACS1*, *PsACS4*, *PsACO1*, *PsERS1*, *PsETR1*, *PsETR2*, *PsERF1a*, *PsERF11* and *PsERF12* are the key genes involved in ethylene-mediated reddening in the flesh of postharvest ‘Friar’ plum fruit under storage at 10 °C.

## Data Availability

The data presented in this study are available within the article.

## Supplementary Materials

The following supporting information can be downloaded at: https://www.mdpi.com/article/10.3390/plants12040893/s1, Table S1: alignment of homologous genes selected from the transcriptome data; Table S2: the sequence of primers of for qRT-PCR.
