# Supplementary material for: Ethylene Signal Is Involved in the Regulation of Anthocyanin Accumulation in Flesh of Postharvest Plums (Prunus salicina Lindl.)
Source: Plants (Basel). 2023 Feb 16;12(4):893. doi: 10.3390/plants12040893 (PMC9963230; doi:10.3390/plants12040893)
Supplement: Supplementary file 1 [file plants-12-00893-s001.zip › Supplementary Table S1 Alignment of homologous genes selected from the transcriptome data.pdf]

Table S1 Alignment of homologous genes selected from the transcriptome data

Gene	Number	Identity
ACS4	5	97.88%
ACO1	4	97.12%
ERS	5	91.79%
ETR1	3	98.53%
ETR2	3	97.53%
EIN4	4	72.72%
CTR1	6	56.11%
EIN3/EIL1	11	76.75%
ERF1a	4	89.84%
ERF12	3	99.29%
