# Supplementary material for: Ethylene Signal Is Involved in the Regulation of Anthocyanin Accumulation in Flesh of Postharvest Plums (Prunus salicina Lindl.)
Source: Plants (Basel). 2023 Feb 16;12(4):893. doi: 10.3390/plants12040893 (PMC9963230; doi:10.3390/plants12040893)
Supplement: Supplementary file 1 [file plants-12-00893-s001.zip › Supplementary Table S2 The sequence of primers of for qRT.pdf]

Table S2 The sequence of primers of for qRT-PCR.

Gene	Accession no.	Forward	Reverse
PsACT	XM_008234719.2	CTGGACCTTGCTGGTCGT	ATTTCCCGCTCAGCAGTG
PsPAL	XM_008245733.2	AATGCAAGCACTTCAATCTTCC	GAACTCCAGCACTTCCACTATCC
PsCHS	XM_008226357.2	AGAGTCCAAATTAGCCCTGAAGC	AATAAATAGCACACAGGCACTGG
PsDFR	XM_007222255.2	GCCAACAATAAATGGGGTGC	ATTCACGGTTCCTGCTGAGG
PsF3H	XM_007202045.2	GCACAATTACCCTTTTGCTCC	CCCATTGCTCAGAAAATGACC
PsANS	XM_007210458.2	ATTTGGCCTCAAACACCTGC	ATCCCAACCCAAGTGACAGC
PsUFGT	XM_021949698.1	ATGTCGGACCTTTCAACCTAGC	AGGACAGGCAACCAGTAACGTC
PsWIN1	XM_007216691.2	ACCCCAGCCAGAAACTAGCA	TGCAACCCTTTCCTCGTCAT
PsMYB10	Pd.00g623010	AATAAGACCTCAACCCCGAAGC	TTCCCACCAATCCTTTCCGTTT
PsACS1	Pd.00g1111090	ATTCATCAACCAAAAGAACATCC	CAATTCATGTTTTGTATGACCTCG
PsACS4	Pd.00g1126480	GATCATAACAAACCCATCAAACC	TGCATAGATTTCGTCACACACC
PsACO1	Pd.00g1254750	GCAGAGGTTCAAGGAGCTGG	GCGCAAGTAGAAGGTGCTTT
PsERS1	Pd.00g069100	CCCCTATTAAATCTTTCGAATTTCC	CTGATCTGCAACAACATCAACC
PsETR1	Pd.00g189180	GAATTGATGGTTACGAACTTGCTG	TCCATCCATACCAACCCTCATG
PsETR2	Pd.00g791340	AAATGATGTGATGGATAATTCTGC	GCCCTTGTACACACACAAGC
PsCTR1	Pd.00g887430	GAATACTACGCTCCGACTCTATCG	TTCACCACCTCCACCAACC
PsEIN2	Pd.00g027930	CCCAGCCAGCCACAATACAT	CACCCAATGAAGAAGCGGA
PsEIN3/EIL1	Pd.00g550550	GCAGCTCCAGAGCATGACC	CTTTAAGTCGCTTCAATAGCATCC
PsERF1a	Pd.00g424390	CTGACGCGGATATCACACTCC	GAGAAAATCGGACCCGTAACC
PsERF11	Pd.00g241640	CCAGAGACGGGAGCAACAAT	GGAACTAACACCCTGAGCGAGA
PsERF12	Pd.00g861270	GCGACTGCTTCTTCATCTCG	ACCAAACCCTGGTCTTCTTCC
